# SARS-CoV-2 vaccination complicated by small fiber neuropathy, mast cell activation syndrome, and pericarditis

**DOI:** 10.1016/j.clinsp.2023.100304

**Published:** 2023-11-11

**Authors:** Danice Hertz, Fulvio Alexandre Scorza, Ana Claudia Fiorini, Josef Finsterer

**Affiliations:** a Gastroenterology Private Office, Santa Monica, CA, USA; b Disciplina de Neurociência, Universidade Federal de São Paulo/Escola Paulista de Medicina (UNIFESP/EPM), São Paulo, SP, Brazil; c Programa de Estudos Pós-Graduado em Fonoaudiologia, Pontifícia Universidade Católica de São Paulo (PUC-SP), São Paulo, SP, Brazil; d Departamento de Fonoaudiologia, Escola Paulista de Medicina/Universidade Federal de São Paulo (EPM/UNIFESP), São Paulo, SP, Brazil; e Neurology & Neurophysiology Centre, Vienna, Austria

## Commentary

SARS-CoV-2 vaccines are not free from side effects. One of them is Small Fibre Neuropathy (SFN).^1^ SFN exclusively affects A-delta or C-fibres and is clinically characterized by pain, sensory disturbances, and autonomic dysfunction.^2^ Mast Cell Activation Syndrome (MCAS) has been reported in association with SARS-CoV-2 infections but not in association with SARS-CoV-2 vaccines.^3^ MCAS is characterized by repeated episodes of anaphylactic symptoms such as hives, swelling, low blood pressure, difficulty breathing, or severe diarrhea. During these episodes, large amounts of mast cell mediators are released.^4^ Another known complication of SARS-CoV-2 vaccines is pericarditis.^5^ Pericarditis often occurs together with myocarditis and can be complicated by heart failure and malignant ventricular arrhythmias. Here the authors report the first patient with SFN, MCAS, and pericarditis shortly after vaccination with an mRNA-based SARS-CoV-2 vaccine.

The patient is a 66yo previously healthy female who developed facial tingling, oral numbness, blurred vision, dizziness, and an elevated blood pressure of 176/127 mmHg, 30 min after receiving the first dose of the BNT162b2 vaccine (Biontech Pfizer Vaccine [BPV]) with the lot (batch) number EK5730 in 12/2020. One day later, she developed severe dysesthesias of the entire integument (“burning skin”), predominantly of the face and head, as well as diffuse numbness, tingling, twitching, electrical feelings throughout, internal vibrations, tinnitus, blurred vision, tremors, trouble speaking, profound weakness, post-exertional malaise and brain fog. She also experienced a tight band-like constriction around the chest, left-sided chest pain, pounding tachycardia, and shortness of breath. She noted watery diarrhea for the first two weeks followed by severe constipation. She also experienced dysautonomia manifested by dizziness, imbalance, Postural Tachycardia Syndrome (POTS), urinary hesitancy, and profuse sweating. Heart rate increased by 25‒30 points when standing. She also noted excessive hair loss. Anti-ACE-2 antibodies were elevated at 13.7 (n, < 9.8 U/mL). Nerve Conduction Studies (NCSs) and needle Electromyography (EMG) were inconclusive. Skin biopsies demonstrated normal Intra-Epidermal Nerve Fibre Density (IENFD). Anti-TSHDS-IgM and anti-FGFR3 antibodies were negative. SFN was diagnosed based on the clinical presentation and normal NCSs. Gabapentin (GPT) was started (maximum 1200 mg/d) and initially had no impact on the neuropathic pain. Cetirizine was started but was replaced by loratidine (maximum 10 mg/d). She was also started on famotidine 40 mg/d, propranolol 30 mg/d, and various vitamins and supplements. Three courses of steroids were ineffective. Subcutaneous Immunoglobulins (SCIGs) caused an increase in neuropathic pain and were discontinued after three months. Because levels of tryptase, chromogranin-A, and urinary prostaglandin F2-alpha were elevated, MCAS was diagnosed. Tryptase levels ranged from 13.3‒17.7 mcg/L with a median value of 15 mcg/L (n, < 11 mcg/L). She tested negative for the Kit D816 mutation and for mutations in the Hereditary Alpha-Tryptasemia (HAT) genes. Oral cromoglicic acid (800 mg/d) was started without improvement. She followed a low histamine diet and used Diamine Oxidase (DAO) supplements with meals. A few days after stopping the SCIGs, the left-sided chest pain increased, and because echocardiography revealed a moderate pericardial effusion, pericarditis was diagnosed, and colchicine (maximum 1.2 mg/d) and low dose ASA 81 mg/d were started with improvement in the chest pain. Westergren sedimentation rate, C-reactive protein, and autoimmune panel were all normal. D-dimer and troponins were also normal. Total complement was persistently high at > 60 U/mL (n, 31‒60 U/mL). Alanine-Transaminase (ALT) was intermittently elevated to 105 U/L (n, 6‒55 U/L). Extensive hepatology evaluation was negative. She was found to have a small paraprotein migrating as IgA with lambda light chains on serum immunofixation. As of 4/2023, this was no longer present. Flow cytometry lymphocyte panel revealed a CD4:CD8 ratio of 12. This normalized to 3.7 by 2/2022.

Clinical improvement has been very slow. After 16 m, a Low Dose Naltrexone (LDN) (maximum 1.2 mg/d) was started as well as time-restricted eating (eating in a 6-hour window each day) and some additional improvement was noted, but symptoms of SFN, MCAS, and pericarditis have not fully resolved as of the last follow-up visit in 7/2023. She is currently taking GBT (300 mg/d), propranolol (30 mg/d), loratidine (10 mg/d), famotidine (20 mg/d), colchicine (0.3 mg/d), acetyl-salicylic acid (81 mg/d), naltrexone (1.2 mg/d), and cromoglicic acid (200 mg/d) as well as multiple vitamins and supplements.

SFN has been repeatedly reported as a complication of SARS-CoV-2 vaccines. However, a causal relationship between vaccination and SFN is often questioned. This happens most often with studies sponsored by manufacturers or those who are convinced that SARS-CoV-2 vaccinations cause no or only mild side effects. Notwithstanding this attitude, there is mounting evidence that SARS-CoV-2 vaccines can cause serious side effects in some cases.

SFN after BPV was reported in a 40yo female with severe fatigue, dizziness, flushing, palpitations, diarrhea, muscle weakness, and gait disturbances.^6^ The skin punch biopsy showed reduced IENFD.^6^ SFN after BPV was also reported in a 32yo female with severe fatigue, brain fog, dizziness, pre-syncopal sensations, hair loss, chest pain, shortness of breath, palpitations, paresthesias, irregular menstrual cycles, muscle weakness, and hives one day after the second dose of BPV.^6^ SFN was confirmed by reduced IENFD.^6^ SFN was also diagnosed in a 52yo male with paresthesias and burning and stabbing pains in the arms, face, and eyes, accompanied by high‐pitched tinnitus in the right ear.^7^ He subsequently developed orthostatic intolerance and was unable to stand and walk without syncope. These symptoms lasted for more than 5 months.^7^ To our knowledge, MCAS has not been reported after BPV but pericarditis or myocarditis is a known complication of BPV in children and adults.^8^ Whether BPV worsens pre-existing SFN is unknown, but other SARS-CoV-2 vaccines are known to be safe in MCAS patients.^9^ However, in a retrospective study of ten patients who received BPV, vaccination was complicated by spontaneous urticaria.^10^

This case demonstrates that vaccination with BPV can occasionally be complicated by SFN, MCAS, and pericarditis. New symptoms after SARS-CoV-2 vaccination must be taken seriously and require thorough investigation. The earlier complications of SARS-CoV-2 vaccination are detected, the earlier treatment can be started and the more favorable the outcome.

## CRediT authorship contribution statement

**Danice Hertz:** Investigation, Validation, Writing – review & editing. **Fulvio Alexandre Scorza:** Investigation, Validation, Writing – review & editing. **Ana Claudia Fiorini:** Investigation, Validation, Writing – review & editing. **Josef Finsterer:** Conceptualization, Data curation, Formal analysis, Investigation, Methodology, Supervision, Validation, Visualization, Writing – original draft.

## Declaration of Competing Interest

The authors declare that they have no known competing financial interests or personal relationships that could have appeared to influence the work reported in this paper.
